# Carbuncles and Boile

**Published:** 1854-11

**Authors:** 


Carbuncles and Boile.—The Dublin Medical Press contains some proper remarks in regard to the efficacy of lead plasters—the common lead or litharge plaster spread upon soft white leather—in these painful and troublesome affections, which have prevailed in some places epidemically during the past year or two. After poultices and fomentations in the active stage of the inflammation, it is advised to apply a lead plaster so as to cover the tumor and extend a little beyond it upon the sound skin. The effect is to stimulate the exhalent vessels to an increased discharge of perspirable matter, allay irritation and pain, and hasten absorption and suppuration. When the latter exists, an opening in the plaster should be left to facilitate the discharge of matter, and the plaster should be changed only as may be necessary for cleanliness. In this account nothing is said of the more stimulating applications, iodine, alcohol, camphor, &c., to facilitate the resolution of the tumor. The two plans might be combined with great promise of increased advantage.—*Memphis Med. Rec.*

